# Prehospital Electronic Patient Care Report Systems: Early Experiences from Emergency Medical Services Agency Leaders

**DOI:** 10.1371/journal.pone.0032692

**Published:** 2012-03-05

**Authors:** Adam B. Landman, Christopher H. Lee, Comilla Sasson, Carin M. Van Gelder, Leslie A. Curry

**Affiliations:** 1 Department of Emergency Medicine, Brigham and Women's Hospital and Harvard Medical School, Boston, Massachusetts, United States of America; 2 Department of Emergency Medicine, Yale University, New Haven, Connecticut, United States of America; 3 Department of Emergency Medicine, University of Colorado School of Medicine, Denver, Colorado; 4 Robert Wood Johnson Foundation Clinical Scholars Program and School of Public Health, Yale University, New Haven, Connecticut, United States of America; University of Edinburgh, United Kingdom

## Abstract

**Background:**

As the United States embraces electronic health records (EHRs), improved emergency medical services (EMS) information systems are also a priority; however, little is known about the experiences of EMS agencies as they adopt and implement electronic patient care report (e-PCR) systems. We sought to characterize motivations for adoption of e-PCR systems, challenges associated with adoption and implementation, and emerging implementation strategies.

**Methods:**

We conducted a qualitative study using semi-structured in-depth interviews with EMS agency leaders. Participants were recruited through a web-based survey of National Association of EMS Physicians (NAEMSP) members, a didactic session at the 2010 NAEMSP Annual Meeting, and snowball sampling. Interviews lasted approximately 30 minutes, were recorded and professionally transcribed. Analysis was conducted by a five-person team, employing the constant comparative method to identify recurrent themes.

**Results:**

Twenty-three interviewees represented 20 EMS agencies from the United States and Canada; 14 EMS agencies were currently using e-PCR systems. The primary reason for adoption was the potential for e-PCR systems to support quality assurance efforts. Challenges to e-PCR system adoption included those common to any health information technology project, as well as challenges unique to the prehospital setting, including: fear of increased ambulance run times leading to decreased ambulance availability, difficulty integrating with existing hospital information systems, and unfunded mandates requiring adoption of e-PCR systems. Three recurring strategies emerged to improve e-PCR system adoption and implementation: 1) identify creative funding sources; 2) leverage regional health information organizations; and 3) build internal information technology capacity.

**Conclusion:**

EMS agencies are highly motivated to adopt e-PCR systems to support quality assurance efforts; however, adoption and implementation of e-PCR systems has been challenging for many. Emerging strategies from EMS agencies and others that have successfully implemented EHRs may be useful in expanding e-PCR system use and facilitating this transition for other EMS agencies.

## Introduction

### Background and Importance

The Emergency Medical Services (EMS) system provides out of hospital emergency care to patients with traumatic injuries and medical emergencies from initial 9-1-1 call to dispatch, field response, transport, and handoff to emergency department (ED) staff. A growing body of evidence indicates that high quality EMS care improves patient outcomes. [1], [2] Similar to ambulatory and hospital-based providers, emergency medical technicians and paramedics perform patient assessments and treatment and are required to document their encounters. EMS crews can provide important information to ED staff during patient handoff, such as initial vital signs and the events leading up to the ED visit for unresponsive or confused patients. [3] However, if this handoff is not received in real-time, ED clinicians must track down paper-based run sheets, which can be hard to locate and even more difficult to read.

Electronic patient care reports (e-PCR) systems have the potential to improve EMS record availability and legibility for ED clinicians, as well as to improve quality assurance, outcomes research, and billing for EMS agencies. [4]–[6] Current commercial e-PCR systems primarily replicate existing paper-based patient care reports with electronic fields to capture history, physical exam, assessment, and treatment rendered. A variety of commercial electronic software and hardware solutions are available and each EMS agency or region generally selects their own product. Formal e-PCR system workflow studies have not yet been completed and EMS providers may use these systems in many different ways. e-PCR system software is typically loaded on rugged tablet computers that EMS providers take with them in the field to capture data in real-time as the call progresses; other EMS providers record handwritten notes during the call and complete electronic reports when they arrive back at their station after call completion. Once finished, the e-PCRs can be provided to the receiving hospital using several possible processes, such as printing e-PCRs in the ambulance or hospital and leaving the hardcopy with the patient's ED records; or faxing the e-PCR to the hospital after report completion.

Many EMS agencies currently use paper-based patient care reports and are transitioning to electronic patient care reports. Improved EMS information systems and integration with other electronic health care records was identified as a national priority in the 1998 Emergency Medical Services Agenda for the Future, the 2006 Institute of Medicine report, EMS at the Crossroads, and most recently at the 2010 Academic Emergency Medicine Consensus Conference. [7]–[9] However, current United States (US) federal legislation and policies incentivizing the adoption of health information technology do not provide support for EMS agencies seeking to move to e-PCR systems or to integrate e-PCR systems with ED or hospital information systems. [10], [11]

The US-based National EMS Information Systems (NEMSIS) project has been a leader in prehospital information systems, developing a standardized data dictionary to represent key EMS data fields as well as a national database where participating states can send their records for research and storage. [4], [5] Although one of the goals of the NEMSIS project is to “implement an electronic EMS documentation system in every local EMS system,” NEMSIS' initial focus has been at the national and state levels. [12] However, the potential value of e-PCR systems at the hospital and regional levels is limited if only a small number of EMS agencies adopt and use e-PCR systems. Research has shown that complex technical, organizational, financial, and privacy/security factors make adoption of healthcare information technology (HIT) difficult in hospital and physician office settings. [13]–[15] It remains unclear whether EMS agencies face similar challenges when moving to e-PCR systems.

### Goals of this Investigation

A 2006 study estimated that less than half of US states collect a majority of EMS incident data electronically, [16] but there has been no work to date examining e-PCR system use at the EMS agency level. As a first step in understanding EMS agency adoption and use of e-PCR systems, we sought to characterize: (1) motivations for adoption of e-PCR systems; (2) challenges with adoption and use of e-PCR systems; and (3) reported strategies for implementation from early adopters of e-PCR systems.

## Methods

### Study Design

We performed a qualitative study using in-depth interviews with key informants, individuals representing multiple levels of the EMS system, including local, front-line EMS agencies and, administrative EMS agencies. [17],[18] We chose a qualitative approach because it is well suited to studying processes of organizational change, [19], [20] such as early experience in adoption and use of e-PCR systems. Given the individual variation in EMS system design and use of technology, one-on-one interviews enabled exploration of a broad range of issues surrounding e-PCR system use across many EMS systems.

### Selection of Participants

Study participants were identified in three ways: 1) a web-based survey distributed to all National Association of EMS Physicians (NAEMSP) members; 2) an announcement at the 2010 NAEMSP Annual Meeting; and 3) recommendations from other participants (snowball sampling). [20] NAEMSP is the leading EMS professional organization with a diverse membership, including physicians, paramedics, nurses, administrators, and educators. [21] As leaders of state, regional, or local EMS agencies, these individuals are intimately familiar with medical record operations, including the acquisition and use of e-PCR systems.

Interviews were completed with the initial wave of volunteers (n = 13) recruited from the web-based survey and didactic session. The web-based survey was developed by the study authors and distributed to all NAEMSP members via electronic mail before the 2010 NAEMSP Annual Meeting. A copy of the survey is provided in Figure S1. It included general questions about the respondent and the EMS agency or agencies they represented as well as specific questions on whether or not they had e-PCR systems, barriers to adoption, and e-PCR system features. Respondents were also asked if they would be willing to discuss their experiences with e-PCR systems in more detail (this study) and were provided a link to e-mail the primary study author if interested. One of the study authors (CVG) presented a didactic session on e-PCR systems at the 2010 NAEMSP Annual Meeting and announced this study and recruited participants at the end of the presentation.

We also used snowball sampling, in which study participants nominated other potential respondents, to complete the sample. [20] Since none of the identified EMS agencies were able to exchange electronic data with hospital electronic medical records (there are very few organizations currently with this functionality), [6] we included a hospital-based emergency physician to represent an organization with this capability. We used a purposeful sampling approach to ensure a balance of geographic locations (Northeast, Midwest, South, West, and Canada) as well as agencies using e-PCR systems and not using e-PCR systems. [20] Data collection and analysis were conducted simultaneously; we continued to recruit participants until thematic saturation was achieved, or no new ideas surfaced from subsequent interviews. [22], [23] A total of 23 EMS leaders, representing 20 EMS agencies, participated in the study. Three participants were randomly selected for $50 gift certificates for their participation in this study.

### Ethics Statement

The study's purpose, voluntary nature of participation, and confidentiality of responses were written in the web-based survey preamble (Figure S1). Survey participants were offered the opportunity to provide their contact information if they were willing to be contacted to participate in the in-depth interview. The same study information was verbally described to potential participants identified during the NAEMSP Annual Meeting announcement and through snowball sampling.

Prior to beginning the qualitative interviews, the purpose and plan for the interview, the voluntary nature of the interview, the confidentiality of the interview responses, and whom to contact with questions was again reviewed with all participants. The study, including the informed consent procedures, was approved by the Yale University School of Medicine Human Investigation Committee (Institutional Review Board). To maintain confidentiality of responses, no individually identifiable records were kept.

### Methods and Data Collection

In-depth interviews were conducted between January and April 2010 by two investigators (AL and CL), both emergency physicians with fellowship training in health services research. Each interview lasted approximately 30 minutes and was scheduled at a convenient time for the participant during the 2010 NAEMSP Annual Meeting or after the meeting via telephone. We developed a standard interview guide with five broad questions and probes to encourage clarification and elaboration on specific aspects of e-PCR experiences (Table 1). Interviews were audio recorded and professionally transcribed. Missing text from initial transcriptions was reviewed with the original recordings and re-transcribed by the primary author.

**Table 1:** Qualitative Interview Guide.

**1. Let's start by having you describe the EMS agency(s) that you are affiliated with and your roles and responsibilities.** Provide comfortable, non-threatening way into the interview; begin to establish a relationship; understand the organization the person works within and their roles; and gain a sense of their specific role with e-PCR
**2. How does your agency currently document prehospital patient encounters? Can you walk me through the process?** Elicit descriptions of prehospital processes for documentation (paper-based, electronic, or some combination). Describe interactions among dispatch, prehospital, and hospital systems and providers.
**3. Have there been efforts to move to e-PCR in your agency?** What got it started? What challenges arose? How did the organization recognize problems or opportunities? How did you deal with setbacks? Can you describe things that needed to get ironed out along the way?
**4. How does your prehospital agency provide handoff documentation to the ED staff?** Encourage respondent to describe the current process of handoff from prehospital to ED providers. What documentation is exchanged and at what time? How well is this process working?
**5. How has the addition of e-PCR (or lack of e-PCR) impacted your organization?** Do you see value added from the adoption of e-PCR? Alternatively, has e-PCR had deleterious effects on your operations? How has e-PCR affected efficiency and quality of care? Please cite specific examples

### Data Analysis

We applied the principles of grounded theory, [19], [20], [22], [24], [25] including iterative data collection and analysis and the constant comparative method, to inductively generate insights grounded in the experiences of participants.

Each transcript was read and independently coded by three investigators (AL, CL, and CS) under the guidance of senior authors, one with expertise in qualitative methods (LC) and the second with expertise in emergency medical services (CVG). Each transcript was discussed, line-by-line, and consensus reached on each code. Codes were revised, added, and deleted as we progressed through the transcripts. We used an iterative process of data collection and analysis to generate and refine the code structure. We also maintained an audit trail to document analytic decisions. The primary author reviewed all transcripts after the final coding structure was established to ensure all quotes were coded consistently. We then developed key themes, or recurrent and unifying ideas using the approach described by Bradley *et al*, to characterize participant views about reasons for as well as facilitators and impediments to adoption and use of e-PCR systems. [26] Atlas Ti software (v5.6.3, Scientific Software Development, Berlin, Germany) facilitated data organization and retrieval.

As recommended by experts in qualitative analysis, we conducted participant conformation, in which participants reviewed a summary of the data to ensure accurate representation of their views; there were no suggestions for refinement of the findings. [24]

## Results

Twenty-three study participants represented 20 EMS agencies from across the United States and Canada and included medical directors, EMS fellows (emergency physicians undergoing subspecialty training in EMS), and EMS agency administrators (i.e., chief, other officer, training and quality assurance staff) (Table 2). The majority of EMS agencies (14/20) were currently using e-PCR systems. Participants represented different levels of the EMS system, including local, front-line EMS agencies (which provide direct patient care), administrative EMS agencies (i.e., county or state EMS agencies which provide administrative oversight), and a hospital (Table 2).

**Table 2:** Characteristics of Participants (n = 20 EMS Agencies).

	*Number*
**EMS Agency using e-PCR systems**	14
**Respondent Position** *	
Medical Director	10
EMS Fellow	4
Administrator+	8
Other Physician	1
**Type of Agency**	
Local EMS Agency^1^	15
Administrative^2^	4
Hospital	1
**Region**	
Northeast	5
Midwest	6
South	4
West	3
Canada	2

* 3 agencies had 2 persons participating in the interview simultaneously; therefore, for 20 EMS agencies there were 23 respondents.

+ EMS agency administrators such as chief, other officer, training and quality assurance staff.

1 Local EMS agencies provide direct patient care.

2 Administrative EMS agencies provide administrative oversight (i.e., county or state EMS agencies).

Our analysis generated insights into EMS agency motivations for adoption of e-PCR systems and their experiences (both challenges and strategies) across the information systems life cycle from pre-implementation through to maintenance of the mature system. An overview of the themes and subthemes is presented in Table 3 and the themes are described in more detail below.

**Table 3:** Summary of Categories and Themes.

*Motivations for adoption of e-PCR systems*	*Challenges to adoption and use of e-PCR systems*	*Strategies for e-PCR system adoption and use*
Improves quality assurance	Face financial, organizational, technical, and privacy challenges common to many health information technology projects	Identify alternative, creative funding sources
Improves billing and legibility of charts	Fear of increased ambulance run times	Leverage existing regional health information organizations
Decreases lost charts	Difficulty electronically integrating e-PCR records with existing ED/hospital information systems	Invest in internal information technology capacity
Response to state mandate	Difficulty responding to unfunded state mandates requiring adoption of e-PCR systems	

### Motivations for Adoption

Participants cited improved legibility and billing, fewer lost charts, and mandates requiring adoption as reasons for moving to e-PCR systems. However, the potential of e-PCR systems to support quality assurance was the primary motivator for adoption:

“It was virtually impossible for us as a regulatory oversight agency to fulfill our statutory and ethical responsibilities to monitor the quality of EMS using old style paper run reports … and manual data or manual review of records and that kind of thing. We had to do something better.”

[ID#17, County EMS Medical Director, Midwest]

Many participants emphasized that doing quality assurance work via paper-based records is very difficult, and therefore was often not performed:

*“Paper quality assurance is a nightmare. We just don't do it*.” [ID#12, EMS Medical Director, Midwest]

Participants anticipated e-PCR systems would facilitate quality assurance by increasing availability of records and automating reporting on quality metrics, such as intubation success rates and out of hospital cardiac arrest survival rates.

### Challenges to Adoption

Participants expressed numerous barriers to adoption of e-PCR systems. Many of these reported challenges are similar to previously described barriers to HIT adoption in other health care settings. [13], [14] A 2006 Robert Wood Johnson Foundation report categorized these barriers as financial, organizational, technical, and privacy factors. [13] Table 4 summarizes quotes from our participants expressing similar challenges with e-PCR system adoption along these four dimensions: financial (high start-up costs and lack of financial resources); organizational (lack of leadership and complex organizational structures); technical (poor user interface design and unreliable vendors); and privacy (concerns about privacy and security).

**Table 4:** Challenges to e-PCR system adoption fit within existing models of barriers to health information technology projects.

*Category* 1 */Example Themes* 2	*Sample Participant Quotes*
**Financial**	
High start-up costs	*“I think the main barrier; to be perfectly honest with you is the initial capital expense.”* [ID#12, Medical Director, Midwest]
Lack of financial resources	*“You know, number one, where are you getting the money to do this? … That requires you to have computers. That requires you to have the software in place to do it. That requires you to pay for training. That requires you to pay for backfill training.”* [ID#18, Medical Director, Midwest]
**Organizational**	
Lack of leadership	*“The chief is a short-timer at this point and I don't think he wants to take on anything that will rock the boat or cause him any extra work or trouble.”* [ID#10, Medical Director, Midwest]
Complex organizational structures	Unions or prehospital providers could also delay the process: *“you inevitably have some grinding on the way whether it's the union or the provider who doesn't understand why you're doing what you're doing.”* [ID#13, Administrator, Canada]
**Technical**	
Poor user interface design	*“user interface was a real big thing”* [ID#6, Medical Director, West]
Unreliable vendors	*“We weren't very pleased…they [vendors] were always… “we are going to work on that”…it never happened and then finally it either got bought out by another company or went bankrupt, and that was that.”* [ID#1, Medical Director & Administrator, South]
	*“For example, when Medicare was requiring patient signatures for reimbursement issues…The vendor completely fell behind and we went 3 months without signatures.”* [ID#10, Medical Director, Midwest]
**Privacy/Security**	
Concerns about privacy and security	*“… as hospitals go electronic and EMS goes electronic, how do you transfer the report if not by email, how do you do it in a secure way that doesn't violate HIPAA?”* [ID#7, State Medical Director, South]
	*“… the security that's necessary for … some of the barriers that are pretty large right now. And we're sort of struggling with that at the hospital…”* [ID#5, Medical Director, Northeast]
	*“Their [Hospital] IT people are naturally concerned about the possibility of breaching firewalls and causing more problems by importing the data directly.”* [ID#8, State Medical Director, Northeast]

1 (2006) Health Information Technology in the United States: The Information Base for Progress. Robert Wood Johnson Foundation.

2 Boonstra A, Broekhuis M (2010) Barriers to the acceptance of electronic medical records by physicians from systematic review to taxonomy and interventions. *BMC Health Serv Res* 10: 231.

Study participants also expressed adoption challenges more specific to EMS agencies and e-PCR systems, including: 1) fear of increased ambulance run times; 2) difficulty integrating e-PCR systems with existing ED or hospital information systems; and 3) difficulty responding to unfunded mandates requiring adoption of e-PCR systems. We present a description of each of these challenges with exemplar quotations from study participants below.

#### Increased run times due to e-PCR system adoption

Participants expressed concerns that e-PCR systems would require greater time to complete than paper forms, resulting in increased ambulance run times. One EMS officer from the Northeast described his department's persistent concerns with the impact of extended times to complete reports, even after a substantial amount of experience with e-PCR systems:

*“I'm hearing that even after people have, you know, 100 or 200 electronic reports under their belt, it's still taking them half an hour. Which we didn't want to do to them [referring to not wanting to add documentation time to the EMS crews]. But that's what some of our guys are afraid of.”* [ID#19, EMS Officer, Northeast]

Respondents were concerned that longer run times could lead to ambulances being out of service for greater periods of time, and therefore not be available for the next emergency call. A West Coast EMS Fellow reported that the increased time needed to complete e-PCR systems may have potential negative implications for timeliness of patient care:

*“The increased amount of time that's required to fill out an electronic patient care report has a huge impact on their ability to provide care to their patients. In other words, they aren't able to see as many patients and complete their electronic patient care reports during their shift.”* [ID#3, EMS Fellow, West Coast]

Despite these concerns, some participants observed run times return to baseline levels after their providers adjusted to the e-PCR systems, suggesting increased run times may decline after an initial transition period:

“…from my own personal experience, it was slow when we transitioned to a completely electronic, however it's much faster after I've been doing this for a year or two.”

[ID#18, EMS Medical Director, Midwest]

#### Lack of integration of e-PCR systems with existing ED or hospital information systems

Participants agreed that e-PCR systems should be electronically integrated with ED or hospital information systems. Participants universally experienced difficulty achieving this goal due to technical barriers such as lack of interfaces between e-PCR systems and hospital EMR systems and gaps in mobile broadband coverage as well as organizational issues such as high costs and security/privacy concerns. A majority of agencies resorted to printing hard copies of e-PCRs in the ambulance or ED to provide ED handoff documentation. A Northeast medical director recounted frustration with not being able to seamlessly electronically link e-PCR systems and hospital information systems:

“Everyone's using a different system. …none of the systems interface with each other as well as we'd like them to be. … And so we're left with … great electronic PCRs and they do excellent, but we still can't interface that electronic PCR with the patient's electronic record… in the hospital. And, that's been problematic for us because we have this tool…this device that, you know, we do great work with, but then we've got to revert back to paper run report and then hand that paper run report off to someone like within our original department.”

[ID#5, Medical Director, Northeast]

#### Lack of funding for state mandated e-PCR system implementation

Prehospital care, like other areas of healthcare, is heavily regulated by an array of federal, state, and local policies. Since e-PCR systems can facilitate data management and analysis, some state EMS agencies with administrative purview have used their statutory authority to mandate adoption of e-PCR systems by EMS agencies within their jurisdictions. Participants representing front-line EMS agencies expressed frustration with these mandates, particularly since many states did not provide any resources to support the necessary software, hardware, and training expenses. The mandates were sometimes accompanied by penalties for noncompliance, including loss of license as described by a Midwest medical director:

“This is the unfunded mandate from the state of [name] EMS division, so the compliance is not as good as they would like it to be. But the ultimate penalty is going to be not having your [state EMS agency] license renewed.”

[ID#18, Medical Director, Midwest]

### Emerging Strategies from the Field

While implementing and sustaining e-PCR systems is difficult, participants also reported lessons learned in implementing their own e-PCR systems and offered suggestions for other agencies. Three recurring strategies emerged: 1) identify creative funding sources; 2) leverage regional health information organizations (RHIO); and 3) build internal information technology (IT) capacity.

#### Identify alternative, creative funding sources

Cost has been identified as a principal barrier to HIT adoption in the literature [13], [14] and was also expressed by participants as a barrier for e-PCR adoption (Table 4). While some EMS agencies supported e-PCR system adoption with internal funds, several agencies found alternative sources of funding, including state/federal grants, billing companies and e-PCR system vendors.

EMS data is also a critical source of data for injury prevention researchers and government transportation safety officials. [27], [28] One state EMS medical director from the Northeast described working with state highway traffic safety officials that were interested in getting access to EMS data.

*“So they [state highway safety office] were willing to put up a sizeable amount of money to help us get this system.”* [ID#8, State EMS Medical Director, Northeast]

Other agencies have worked closely with their billing companies, who subsidize e-PCR systems for their EMS agencies.

*“I think primarily because they [billing company] were having such a difficult time billing off of paper based charts that they found it advantageous to provide this as a service because it improves billing.”* [ID#9, EMS Fellow, Northeast]

Early adopters of e-PCR systems were also able to serve as beta testers for e-PCR vendors and receive discounted or free software in exchange for product feedback.

#### Leverage existing regional health information organizations

A key challenge to the adoption, implementation and utilization of e-PCR systems is linkage with ED or hospital information systems. Three participants reported working with their existing community RHIO to electronically exchange e-PCRs. RHIOs are generally non-profit, multi-stakeholder entities that facilitate health information exchange among health care providers in a defined geographic region. [29] Using this established infrastructure, an EMS agency can make a single electronic interface with the RHIO and exchange information with all participating hospitals and physician offices. This is a particularly valuable information exchange strategy for EMS agencies transporting to multiple facilities. The efficiency of this approach was reiterated by a hospital-based emergency medicine physician from the Midwest:

“You need that sort of RHIO in place. Otherwise, you're setting up connectivity with each individual system, which would be likely a little painful. And then you'd have to disambiguate all the data. So you might get multiple CBCs [complete blood counts] back from the different sources and then you have to figure out which one to display. So you really need to sort of have this sort RHIO already established to be of value I think.” [ID#20, Hospital-based Emergency Physician, Midwest]

#### Build internal IT capacity

As agencies implemented e-PCR systems, they recognized the need for personnel to manage the systems in order to produce quality assurance reports, customize their software, and support EMS providers who would use the systems. While EMS agencies typically do not have existing dedicated IT support personnel, several agencies described the benefits of funding dedicated IT staff. One Northeast EMS Officer shared the benefit of supporting a current emergency medical technician (EMT) or paramedic to work on EMS information systems:

“We have a paramedic who had an aptitude toward the computers. She was willing to put the time in and she is pretty much dedicated now to just dealing with our computers and our computer systems. She [paramedic] has the ability to go in and get to the server and she can change things on the server and she does a phenomenal job programming the software. She has the ability to think from the ground upward instead of the top downward.” [ID#2, EMS Officer, Northeast]

## Discussion

This is the first study to explore the experiences of e-PCR system adoption and implementation at the EMS agency level. We found that EMS agencies are moving to e-PCR systems primarily to improve quality assurance; however, efforts to adopt and use e-PCR systems are constrained by a number of challenges both common to HIT implementation and unique to this setting. While front-line EMS providers are likely to be familiar with these challenges, our results help inform ED, hospital, and HIT leadership about EMS agency IT needs. We also identified specific strategies that may assist EMS agencies with the transition to e-PCR systems.

Previous research has established an extensive taxonomy of financial, technical, organizational, and legal/regulatory factors that influence the adoption of HIT. [13]–[15] Participants in our study expressed many of these same concerns, particularly the financial challenges associated with purchasing and maintaining e-PCR systems and organizational challenges, including lack of leadership, and operating within complex, bureaucratic organizational structures (Table 4). Although addressing these common barriers has been previously reported as important for successful HIT implementation [30], cost was the primary challenge addressed by our participants. Participants did not widely report strategies for handling other shared HIT barriers, such as lack of leadership, complex organizational structures, poor user interface design, unreliable vendors, and concerns about privacy and security. A systematic approach to e-PCR system implementation addressing these additional people, process, and technology issues may improve the chance of a successful transition to e-PCR systems. [30], [31]

Our study also identified unique challenges to HIT adoption in the EMS setting. Study respondents expressed concerns that e-PCR completion would be more time consuming than paper-based records, therefore placing the ambulance out of service for longer periods of time and unavailable to respond to new emergency calls. Ambulatory providers also share concerns that electronic documentation will require additional time. [32], [33] This is particularly important as prehospital care may be more time sensitive than ambulatory care given the urgent need for EMS resources to be immediately available for the next emergency call. [34] Study respondents further expressed difficulty electronically integrating e-PCR systems with existing ED/hospital information systems. Health information exchange across institutions is a national challenge for all health care providers. [35] Real-time information exchange may be more difficult for mobile prehospital providers operating in austere settings lacking robust wireless communication infrastructures. [6] Study participants also reported difficulty responding to mandates requiring the adoption of e-PCR systems. Other US health care providers are being encouraged to adopt HIT under federal health policy, most notably from the Health Information Technology for Economic and Clinical Health (HITECH) Act, which provides incentive funding for hospital and ambulatory EHR adoption (with penalties in subsequent years if not adopted). [11] In contrast, EMS agencies are being mandated to adopt e-PCR systems by their governing bodies (regions, states), few with incentive funding and some facing significant consequences for noncompliance, such as loss of operating licenses. Consequently, while EMS agencies may find some synergies with hospitals and ambulatory practices in developing solutions to these similar kinds of challenges, solutions must be adapted to work in the EMS setting. [14], [15]

Despite widespread concerns about state mandates and increased ambulance run times, many of the participants successfully adopted e-PCR systems. Notably, despite resource constraints, all participants who reported state mandates to implement e-PCR systems had either successfully adopted or were making progress towards implementation. Successful e-PCR system implementers reported using existing resources more efficiently or creatively. These agencies obtained grant funding, established relationships with their billing companies to pay for their e-PCR systems, and trained their existing staff on HIT development/support. Furthermore, despite substantial fear of increased run times, several participants noted that over time, ambulance run times returned to baseline. Future empirical study is necessary to determine the impact of e-PCR systems on ambulance call times and availability.

### Policy Implications

By taking advantage of creative funding sources, leveraging regional health information organizations, and building internal information technology capacity, several EMS agencies we studied have been able to successfully implement e-PCR systems. Other EMS agencies seeking to adopt e-PCR systems can learn from these experiences and from other health care providers that have successfully implemented HIT. The Agency for Healthcare Research and Quality (AHRQ), Health Resources and Services Administration (HRSA), and Office of the National Coordinator for HIT (ONC) disseminate resources and knowledge about HIT implementations for hospitals and physician offices. HRSA's “HIT Adoption Toolkit” provides a collection of resources covering planning, implementation, and evaluation of HIT systems for ambulatory care providers. [36] While these may be helpful for EMS agencies, there are currently no resources addressing the specific needs of EMS agencies adopting e-PCR systems. A similar adoption toolkit for EMS agencies could be created, based on our work and others in the field, and distributed through the NEMSIS Technical Assistance Center or ONC's Regional Extension Centers.

As the United States makes progress implementing HIT in hospitals and physician offices, [37], [38] new policies and resources can be directed towards improving HIT capabilities in other important clinical settings, such as EMS. [6], [9] Our research, combined with a future comprehensive, quantitative survey of US EMS agency e-PCR system capacity as well as barriers and strategies to e-PCR system adoption, implementation and use, could better characterize the unique needs of EMS agencies. Federal support for additional e-PCR systems research could help determine the impact of e-PCR systems on ambulance call times and identify efficient e-PCR system workflows. Recommendations for increasing the meaningful use of e-PCR systems could then be relayed to the US government to promote a multi-pronged effort to increase e-PCR system adoption through policy, financial incentives, and training and support. [11], [39] Future federal and state regional health information exchange policy could include incentives or requirements to include EMS representatives to facilitate prehospital and hospital information exchange.

### Limitations

Our findings should be interpreted in light of several limitations. First, we developed a sample that was diverse in key characteristics, but our findings may not transfer to all EMS agencies. In particular, our findings may not be relevant in developing countries. However, countries starting EMS programs may have a distinct advantage, learning from these experiences and planning for e-PCR system use from EMS system inception. Findings from qualitative studies are not intended to be generalized, but rather to provide insights into areas which have been previously unexplored and to generate hypotheses that may be tested in larger quantitative studies. [40] Although our sample included NAEMSP members, national, state, and local EMS leaders, some participants may not have operational experience using e-PCR systems for patient care. We also did not include hospital IT representatives and therefore cannot discuss their role in e-PCR system integration. Second, social desirability response bias, in which participants may have misrepresented their improvement efforts in order to provide desirable answers, may have occurred. [41] To minimize this, we elicited details that would be difficult to misrepresent, such as specific technical features of e-PCR systems, and we encouraged respondents to share both positive and negative experiences. Third, this was an exploratory qualitative research study, intended to generate hypotheses about potential challenges and strategies for adoption and implementation of e-PCR systems. We highlighted recurring challenges and strategies raised by participants, but did not ask participants to rank the importance of all challenges and strategies offered.

### Conclusion

At a time when HIT is a national priority, EMS agencies are highly motivated to adopt e-PCR systems to support quality assurance efforts. They face financial, organizational, technical, and privacy/security issues that are common to many HIT projects as well as additional challenges that are unique to e-PCR system adoption, including fear of increased ambulance run times leading to decreased ambulance availability, difficulty integrating e-PCR systems with existing ED or hospital information systems, and unfunded mandates requiring EMS agency adoption of e-PCR systems. Attention to these challenges of e-PCR system adoption as well as change management principles, such as strong technical skills, project management skills, and people and organizational skills, may also improve the success and value of e-PCR system implementations. Emerging implementation strategies from hospitals, ambulatory practices, and EMS agencies that have overcome these barriers, including using creative funding sources, leveraging existing RHIOs, and building internal IT capacity, may be of use to EMS agencies transitioning to e-PCR systems. Additional empirical studies of the unique challenges to e-PCR systems adoption as well as efforts to facilitate sharing lessons learned from e-PCR system implementations, possibly with support from federal agencies, are urgently needed.

## Supporting Information

Figure S1 **Web-based Survey.**
(PDF)
