# Supplementary material for: Prehospital Electronic Patient Care Report Systems: Early Experiences from Emergency Medical Services Agency Leaders
Source: PLoS One. 2012 Mar 5;7(3):e32692. doi: 10.1371/journal.pone.0032692 (PMC3293855; doi:10.1371/journal.pone.0032692)
Supplement: Figure S1 — Web-based Survey. (PDF) [file pone.0032692.s001.pdf]

# NAEMSP e-PCR Survey

## 1. NAEMSP e-PCR Survey

In preparation for the January 7, 2010, NAEMSP session on electronic-prehospital care reports (e-PCR), we are surveying NAEMSP members on their use of and experiences with e-PCR systems. We hope this information will inform a more practical discussion of issues that are important to you during the e-PCR session.

This survey contains approximately 10 questions and we estimate it will take about 10 minutes to complete. Please select the single best response, unless instructed otherwise.

No agencies or individuals will be identified. Individual responses will be kept confidential; only aggregate results will be reported.

Thank you in advance for your time and participation.

Carin Van Gelder, MD
Assistant Professor of Emergency Medicine
Medical Director, New Haven Sponsor Hospital Program
Yale University School of Medicine

Adam Landman, MD, MS, MIS
Robert Wood Johnson Clinical Scholars Program
Instructor, Department of Emergency Medicine
Yale University School of Medicine

## 2. About you

1. What is your highest level of clinical training (select single best response)?

- ○ Physician (MD/DO)
- ○ Licensed Independent Practitioner (i.e., PA, APRN)
- ○ EMT-P, EMT-I, EMT-B
- ○ No Clinical Training
- ○ Other (please specify)

2. What is your position (check all applicable)?

- ☐ Medical Director
- ☐ Prehospital Provider
- ☐ Medical Student/Resident/Fellow
- ☐ Administrator
- ☐ Other (please specify)

3. What is your level of involvement with e-PCR systems (check all applicable)?

- ☐ Purchaser/decison maker
- ☐ Responsible for implementation
- ☐ Responsible for funding
- ☐ User for Patient Data Entry
- ☐ User for Quality Assurance
- ☐ Other (please specify)

4. What training in e-PCR have you had (check all applicable)?

- [ ] None
- [ ] Trained by vendor
- [ ] Trained by non-vendor
- [ ] Learned on my own - trial and error
- [ ] Learned on my own - books
- [ ] Other (please specify)

# NAEMSP e-PCR Survey

## 3. About your agency(s)

Since you may participate in or provide medical direction to more than one EMS agency, your answers to the next two questions will help us understand how many agencies you are involved with and their support for e-PCRs.

The * denotes that responses to these questions is required.

* 1. For how many prehospital agencies are you involved as a provider, administrator, or medical director?

* 2. What percentage of your agencies currently support e-PCR?

- ◯ 0
- ◯ 25
- ◯ 50
- ◯ 75
- ◯ 100

## 4. Agencies with NO support for e-PCR

The next two questions are designed for agencies that do NOT currently support e-PCR.

If your agency currently has e-PCR, please click the Prev button below and change your responses to the previous two questions.

### 1. What are the barriers to your agency's adoption of e-PCR? (select all applicable)

- [ ] Cost
- [ ] Technical Expertise
- [ ] Lack of support from leadership
- [ ] Benefits of e-PCR unclear
- [ ] Other (please specify)

### 2. Do you plan to implement e-PCR in the future? (select single best response)

- ○ in next 6 months
- ○ in 6 - 12 months
- ○ in over 1 year
- ○ No current plans
- ○ Unknown

## 5. Agencies with some e-PCR

These questions are designed for persons who have some agencies with e-PCR capability, but some without e-PCR capability.

If you have NO e-PCR or all your agencies have e-PCR, please click the Prev button at the bottom of the page and change your answers to the previous two questions.

### 1. What are the barriers to your agency's adoption of e-PCR? (select all applicable)

- [ ] Cost
- [ ] Technical Expertise
- [ ] Lack of support from leadership
- [ ] Benefits of e-PCR unclear
- [ ] Other (please specify)

### 2. For your agencies without e-PCR, do you plan to implement e-PCR in the future? (select single best response)

- ○ in next 6 months
- ○ in 6 - 12 months
- ○ in over 1 year
- ○ No current plans
- ○ Unknown

## 6. e-PCR

The following questions are designed for agencies that currently support e-PCR. If your agency currently does NOT support e-PCR, please click the Prev button below and change your responses to the previous two questions.

If you are a member of or supervise multiple prehospital agencies with different policies, please answer the following question for what MOST agencies do.

1. How many distinct e-PCR systems (i.e., unique vendor products) are in use in your agency or agencies?

2. What year did you first begin using your e-PCR system? (Year)
*If you represent multiple agencies, please respond with the Year that represents most of your agencies.*

3. How did you pay for your system (check all applicable sources)?
*If you represent multiple agencies, please respond with the sources that best represent most of your agencies.*

- [ ] Department funds
- [ ] Additional local government funds
- [ ] State funds
- [ ] Grant funding
- [ ] Billing company support
- [ ] Don't know
- [ ] Not applicable (I don't buy the systems, just use them)
- [ ] Other (please specify)

4. Please estimate the costs of your e-PCR system (at a single agency):
*If you represent multiple agencies, please respond with the annual cost for your most representative agency.*

| | No cost | < $10,000 | $10,000 - $50,000 | > $50,000 | Don't know |
|---|---|---|---|---|---|
| Initial, start-up costs | ○ | ○ | ○ | ○ | ○ |
| Annual costs | ○ | ○ | ○ | ○ | ○ |

5. What documentation do you or your providers MOST COMMONLY hand off to the emergency department when patients are turned over to the ED?
*If you represent multiple agencies, please respond with the answer that represents most of your agencies.*

- ◯ Handwritten report, e-PCR never sent to the hospital
- ◯ Handwritten report, e-PCR sent to the hospital at a later time
- ◯ e-PCR completed and given to the ED prior to leaving the hospital
- ◯ No EMS documentation left in the ED at patient hand off
- ◯ Other (please specify)

6. How do you provide your e-PCR to the receiving hospital?
*If you represent multiple agencies, please respond with the answer that represents most of your agencies.*

- ◯ Print copy in ambulance
- ◯ Print copy in hospital
- ◯ Fax to the hospital
- ◯ E-mail to the hospital
- ◯ Immediate network-based electronic transfer to ED or hospital information system
- ◯ Other (please specify)

7. Does your e-PCR system currently support the following features?
*If you represent multiple agencies, please respond with the answers that best represent most of your agencies.*

| | Yes | No | Unknown |
|---|---|---|---|
| Dispatch information electronically sent to e-PCR and automatically included in report | ◯ | ◯ | ◯ |
| ECG/Code summary information added to e-PCR | ◯ | ◯ | ◯ |
| Data fields change depending on chief complaint selected | ◯ | ◯ | ◯ |
| Data fields automatically populated if patient has been entered in system before | ◯ | ◯ | ◯ |
| Ability to read information from magnetic card (e.g., drivers license, health card) | ◯ | ◯ | ◯ |
| Prehospital providers able to access hospital electronic medical record system in ambulance | ◯ | ◯ | ◯ |
| Reporting features to assist medical director with QA/QI | ◯ | ◯ | ◯ |
| Real-time decision support, such as allergy and drug interaction warnings | ◯ | ◯ | ◯ |

## 7. Conclusion

1. Will you be attending the e-PCR session at NAEMSP on January 7, 2010 from 1:45-2:30pm?

- Yes
- No
- Not sure

2. Are there any additional topics that we have not addressed in this survey, that you would like to discuss during the NAEMSP session on e-PCR?

3. Please share any additional comments and/or questions about e-PCR here.

Would you be willing to talk with us more about your experiences with e-PCR?

If you wish to participate, please send an e-mail with your name and preferred contact info to Adam Landman (adam.landman@yale.edu).

Please note that your e-mail response is completely separate from this survey . Your responses to this survey are anonymous and cannot be linked to the contact information you provide via e-mail.

# NAEMSP e-PCR Survey

## 8. End

Thank you for participating in this survey.

We will be sharing the results at the e-PCR session at the NAEMSP Annual Meeting on Thursday, January 7, 2010 from 1:45pm-2:30pm.

Carin Van Gelder
Adam Landman
